# Acute Lysergic Acid Diethylamide Does Not Influence Reward-Driven Decision Making of C57BL/6 Mice in the Iowa Gambling Task

**DOI:** 10.3389/fphar.2020.602770

**Published:** 2020-12-03

**Authors:** Lauri V. Elsilä, Nuppu Korhonen, Petri Hyytiä, Esa R. Korpi

**Affiliations:** Department of Pharmacology, Faculty of Medicine, University of Helsinki, Helsinki, Finland

**Keywords:** lysergic acid diethylamide, psychedelic, hallucinogen, Iowa gambling task, decision-making, executive functions, 25CN-NBOH

## Abstract

While interest in psychedelic drugs in the fields of psychiatry and neuroscience has re-emerged in recent last decades, the general understanding of the effects of these drugs remains deficient. In particular, there are gaps in knowledge on executive functions and goal-directed behaviors both in humans and in commonly used animal models. The effects of acute doses of psychedelic lysergic acid diethylamide (LSD) on reward-driven decision making were explored using the mouse version of the Iowa Gambling Task. A total of 15 mice were trained to perform in a touch-screen adaptation of the rodent version of the Iowa Gambling Task, after which single acute doses of LSD (0.025, 0.1, 0.2, 0.4 mg/kg), serotonin 2A receptor-selective agonist 25CN-NBOH (1.5 mg/kg), d-amphetamine (2.0 mg/kg), and saline were administered before the trial. 25CN-NBOH and the three lowest doses of LSD showed no statistically significant changes in option selection or in general functioning during the gambling task trials. The highest dose of LSD (0.4 mg/kg) significantly decreased premature responding and increased the omission rate, but had no effect on option selection in comparison with the saline control. Amphetamine significantly decreased the correct responses and premature responding while increasing the omission rate. In conclusion, mice can perform previously learned, reward-driven decision-making tasks while under the acute influence of LSD at a commonly used dose range.

## Introduction

During the last 2 decades, there has been a new surge of enthusiasm of psychedelic drugs in the fields of psychiatry and neuroscience (Reiff et al., 2020). This is mainly due to the interesting results from recent clinical studies suggesting that psychedelic drugs may have both rapid and long-lasting therapeutic effects in patients with psychiatric disorders, especially in those with major depression (Carhart-Harris et al., 2016; Griffiths et al., 2016), addiction (Johnson et al., 2014; Bogenschutz et al., 2015), and anxiety-related disorders (Gasser et al., 2014). Concomitant with the clinical studies, a more general interest toward understanding the neurobiological mechanisms of psychedelics has emerged with an increasing body of findings from preclinical animal research (Ly et al., 2018; Hibicke et al., 2020).

While many recently published animal studies have concentrated on models of psychiatric disorders (Alper et al., 2018; Cameron et al., 2018; Hibicke et al., 2020), a more general understanding of the effects of these drugs on the behavior and physiology of the most commonly used animal models remains deficient. This is especially true for the possible effects of psychedelics on executive functions and goal-directed behavior. In healthy human subjects, psychedelics might have some positive effects, as psilocybin has been shown to increase goal-directed behavior toward positive cues compared with negative ones (Kometer et al., 2012). On the other hand, psychedelics are also associated with deficiencies in executive functions, such as disruption of inhibitory processes (Quednow et al., 2012) and with impairments in ﻿Intra/Extra-Dimensional shift task (Pokorny et al., 2019). These data warrant further elucidation of the behavioral effects of psychedelics in animal models of executive function.

The Iowa Gambling Task is a psychological test that was originally developed to simulate real-life decision making in the laboratory environment (Bechara et al., 1994). A commonly used test in clinical psychological research, the task mimics real-life complexity and uncertainty with an in-built unpredictability of the consequences of a choice. In the task, the participant is asked to choose cards, one at a time, from four decks to earn money, with each of the decks having a different probabilistic schedule for monetary gains and losses. This induces a conflict between immediate high rewards and long-term gains. The task is considered to require the integration of several executive functions, like flexibility in planning and evaluation of risk-reward ratio (de Visser et al., 2011). Several slightly different versions of the task have also been developed for rodent experiments (Van Den Bos et al., 2006; Rivalan et al., 2009; Zeeb et al., 2009). These rodent models are based on similar option selection as the original task, but with appetitive instead of monetary rewards. Despite being unable to fully encompass all of the cognitive aspects of the human version, the rodent models are considered to have translational potential (de Visser et al., 2011; Van den Bos et al., 2014). Nevertheless, to our knowledge there are no published studies reporting the effects of psychedelic drugs or serotonin (5-hydroxytryptamine, 5-HT) 2A-receptor (5-HT_2A_) agonists in the rodent versions of the task.

Here, we explored the effects in mice of acute administration of the classic psychedelic drug lysergic acid diethylamide (LSD). We focused particularly on goal-directed decision making while performing in the mouse version of the Iowa Gambling Task based on a design previously reported by Peña-Oliver et al. (2014).

## Materials and Methods

### Subjects

A total of 15 male C57BL/6JRj mice (Janvier Labs, Le Genest-Saint-Isle France) 8 weeks of age at arrival were tested in the Iowa Gambling Task. A group of eight male mice (C57BL/6JCrl in-house bred, originally from Charles River, Wilmington, MA, USA) aged 38–41 weeks at the time of the experiment were tested in the head twitch response experiment. The mice were housed in pairs in individually ventilated cages (GR500, Tecniplast, Buguggiate, Italy) in a temperature-maintained facility with an ordinary 12-h day-night cycle (lights off at 6 p.m.). The cages were provided with aspen bedding, nesting material, a plastic in-cage house, and a piece of wood. Except for the food restriction phase described later, basic rodent chow (Teklad, Envigo, Huntingdon, United Kingdom) and water were freely available. All experiments were conducted between 9 a.m. and 12 p.m. The animal tests were approved by the Animal Experiment Board in Finland (permission no. ESAVI/1172/04. July 10, 2018) and conducted in accordance with national and EU-level ethical and procedural guidelines.

### Iowa Gambling Task Test Apparatus

The test apparatus consisted of six touchscreen-based operant chambers (Med Associates Inc., Fairfax, VT, United States) with a steel grid floor. The chambers were placed inside of wood-composite sound-attenuating cubicles with ventilation fans. The back wall of each chamber was fitted with the touchscreen, covered with a black masking plate with five parallel rectangular holes guiding the interaction with the screen. Throughout the experiment, only uniform white light with no additional shapes or contrasts was used to illuminate the touchscreen, making each of the panels to appear identical. The front wall of the chamber, opposite to the touchscreen, contained a small hole giving access to the reward magazine, where liquid reinforcer was delivered to a small cup with a 20-ml plastic syringe attached to a pump (Syringe pump PHM-100A, Med Associates Inc. Fairfax, VT, USA). The reward magazine had an infrared photo-cell beam for recognizing the head entries, and a stimulus light to illuminate the hole with white light. A house light was mounted on the wall right above the liquid cup. The chambers were controlled with K-Limbic System software for PC (Med Associates Inc., Fairfax, VT, USA).

### Behavioral Procedures

#### Handling

Five days after arrival, the mice were slowly accustomed to handling by the experimenter. Over 5 days, the mice were exposed to gradually intensive handling, ranging from the experimenter slowly moving a hand in the cage and only slightly coming in contact with the mice to lifting the mice away from the cage with an open palm and letting them freely explore the length of the experimenters arm. The procedure was performed systematically and resulted in the same minimum level of habituation with all mice prior to starting the operant training.

#### Food Restriction and Habituation

Ten days after arrival, the mice were restricted of food to reduce their body weights to 80–90% of their free feeding weight. The mean loss of weight was 12% at the beginning of the training (range: 7.7%–16.5%). Water was normally available at all times.

Before the training, the mice were habituated to the operant chambers by letting them freely explore the chambers for 10 min. In the second habituation session, the plastic top of a 15-ml Falcon tube was filled with 20% sucrose solution to be used as the reinforcer in the experiment, and the mice were allowed to freely explore the chambers for 30 min. For the third habituation session, the sucrose solution was provided in the liquid cup as it would be in the experiment and the exploration time was restricted to 15 min. During these sessions, both the touchscreen and the house light were turned off.

#### Mouse Iowa Gambling Task

The training for the gambling task was divided into three phases. During the first phase, four out of the five rectangular panels on the touchscreen were illuminated and any touch to these panels was rewarded with a single presentation of the reinforcer (20% sucrose solution, approximately 30 μL per presentation). Each training session lasted for 30 min or until the mouse responded to the screen 100 times. The mice were then transferred to the second training phase, as soons as there were at least 40 responses in two consecutive training sessions. In the second training phase, the rules of the first phase were in place, but the mice had to activate each trial separately with a nose-poke into the liquid cup hole that was lit with a white stimulus light. Again, each training session lasted for 30 min or until 100 correct responses. The mice were transferred to the last training phase, when they had achieved at least 40 correct responses in two consecutive sessions. In the third, or the forced choice phase, only one of the four panels was randomly lit at a time after the activation of the trial, forcing the mice to respond to the illuminated panel and thus to learn the reward and punishment contingencies of each panel. The rewards and the punishments were the same as in the actual gambling task phase, described below in detail. The sessions lasted for 30 min or until 100 responses. This phase was continued for 3 days. For both the forced choice and the gambling task phases, two different spatial configurations of reward-punishment contingencies were used to counterbalance possible side preferences: in half of the chambers, the order of choices from left to right was P1, P4, P2, P3, and, in the other half, P4, P1, P3, P2 (Configurations A and B, respectively, see Figure 2A).

During the gambling task phase, each session started with the house light going out and the stimulus light in the reward magazine lighting up. A nose-poke to the magazine started a trial with the panels on the touchscreen becoming illuminated for 10 s after a fixed 5-s interval (inter-trial interval, ITI). Any response on the touchscreen during the 10-s illumination time counted as a correct response. Response during the ITI period, when the panels were not yet lit up, was recorded as a premature response and caused an activation of a 5-s time-out period. During the time-out, the screen would stay off and the house light would light up, and any further response to the screen would restart the 5-s timer. After the time-out, the stimulus light would light up and a new trial had to be initiated with a nose-poke. Failure to respond to the touchscreen during the 10-s illumination was recorded as an omission. Omitted trials did not cause any time-out punishments, but the stimulus light in the reward magazine was lit up right after the previous trial ended. If the mouse touched one of the illuminated panels on the screen, the response was recorded as a correct one and the mouse was rewarded or punished depending on the contingencies of the chosen panel. In a rewarded trial, the panels were turned off and the animal received one, two, three, or four presentations of sucrose solution to the reward magazine (P1, P2, P3, P4, respectively, Figure 2A), further reinforced with a clicking sound for each presentation of the reward. If the trial ended with a punishment, a time-out period of 5, 10, 30, or 40 s ensued. During the punishment time-out, the chosen panel flashed at a frequency of 0.5 Hz. The reward-punishment contingencies are presented in Figure 2A. Every week, the mice went through a single gambling task session per day for 5 days with a 2-days weekend without sessions. For each session, the total number of initiated trials, the rates of correctly timed, premature and omitted trials, and the rates of each individual option were measured. See Table 1 for more detailed description of the parameters monitored.

**TABLE 1:** Parameters of the Iowa Gambling Task used for the analysis of the data.

Parameter	Description
Total number of trials	The number of trials that were initiated with a nose-poke to the reward magazine
Proportion of correct responses (Correct %)	The correctly timed responses to one of the four cues on the touchscreen in relation to the total number of initiated trials (Correct/Total)
Proportion of premature responses (Premature %)	The responses during the ITI period in relation to the total number of initiated trials (Premature/Total). May reflect motoric impulsivity
Proportion of omission (Omission %)	The trials with no responses after the initiation, in relation to the total number of trials (Omissions/Total). May reflect inattention or amotivation
Proportion of favorable choices (Favorable %)	The number of P1 and P2 choices in relation to the total number of correct responses (P1+P2/Correct)
Proportion of PX choices (PX %)	The number of PX choices (P1, P2, P3, or P4) in relation to the total number of correct responses (PX/Correct)

Notes: Correctly timed, premature, and missed trials were set in proportion to the number of total trials to accommodate the possible changes in the initiated trials.

#### Drugs and Pharmacological Manipulations

LSD (Sigma-Aldrich, St. Louis, MO, USA), 2-([2-(4-cyano-2,5-dimethoxyphenyl)ethylamino]-methyl)phenol hydrochloride (25CN-NBOH; a kind gift from professor Jesper L. Kristensen, University of Copenhagen; Jensen et al. 2017), and d-amphetamine sulfate (Dexedrine; Smith Kline and French Laboratories, Welwyn Garden City, United Kingdom), were dissolved in sterile saline. The drugs were freshly prepared before administration and all injections were performed intraperitoneally with injection volumes of 10 ml/kg. Both d-amphetamine and 25CN-NBOH doses were calculated as free bases.

After the performance of the mice in the gambling task had stabilized (no significant changes in option selection on four consecutive days on day 15, Figure 2B), the effects of LSD on decision making were tested. Four different doses of LSD were tested (0.025, 0.1, 0.2, and 0.4 mg/kg). The highly selective 5-HT_2A_ receptor agonist 25CN-NBOH (1.5 mg/kg) was used to test for receptor-specific effects (Jensen et al., 2017). Saline (vehicle) was used as the negative control, and 2.0 mg/kg of the psychostimulant amphetamine was the positive control (van Enkhuizen et al., 2013). A 7-days wash-out period was left between the drug administrations. The mice were placed into the test chambers immediately after the LSD, 25CN-NBOH, and saline injections, whereas amphetamine was administered 10 min before starting the sessions. The drug treatments were given in random order with all the mice receiving the same treatment on the same day. The LSD doses were chosen to cover the range of the most commonly used doses in the literature (Stasik and Kidwell, 1969; Halberstadt and Geyer, 2013; Kyzar et al., 2016; Alper et al., 2018). The dose of 25CN-NBOH was chosen based on its ability to prominently induce head twitch responses in earlier publications (Buchborn et al., 2018; Halberstadt et al., 2019).

#### Head Twitch Response

As a separate control experiment, a group of mice (n = 8) was tested for the head twitch response, which is a well characterized acute response for serotonergic hallucinogenic drugs (Halberstadt and Geyer, 2013). On 3 separate testing days, each mouse was injected either with saline, 0.1 mg/kg LSD or 1.5 mg/kg 25CN-NBOH intraperitoneally and immediately placed into a 5-L glass beaker with a white plastic plate placed under the beaker for added contrast. A camera (Sony RX100 M4) was placed above the beaker on a tripod and set to video-record for 10 min immediately after the mouse was placed in the beaker. After the recording, the mouse was returned to its home cage and the beaker was cleaned with a moist cloth and 70% ethanol. The videos were analyzed by visually scoring the number of head twitches. A 5-day wash-out period was left between the testing days in order to avoid possible tolerance induced by serotonergic hallucinogens (Buchborn et al., 2015, Buchborn et al., 2018). Each mouse received the same treatment on the same day.

### Data Analysis

The tested parameters of the Iowa Gambling Task are described in Table 1. All data were tested for normality using Shapiro-Wilk’s test. As none of the datasets were perfectly normally distributed, non-parametric Friedman’s analysis of variance, with all the drug doses or days compared to each other as within-subject variables for each tested parameter, was used to analyze the differences in the data from the gambling task experiment. In the case of a significant result in the omnibus test, Dunn’s pairwise comparison with Bonferroni correction was employed for *post hoc* analysis. All data expressed as percentages were transformed using the arcsine transformation ($x^{'}=2\times \text{arcsin}(\sqrt{x})$) before statistical analysis. Differences between drug treatments in the head twitch response test were tested with Friedman’s analysis of variance followed by Dunn’s pairwise comparison with Bonferroni correction. Except for effect size computations, all statistical analyses were performed using SPSS 24 software (IBM, Armonk, NY, USA). Effect size computations were calculated for Dunn’s z values with Psychometrica’s online tool (Lenhard and Lenhard, 2016). The level for statistical significance was set at *p* < 0.05 and effect sizes were interpreted based on Cohen’s limit values (d > 0.2 small, d > 0.5 medium, d > 0.8 large) (Cohen, 1992). The following report on the results is focused on comparison to the saline control, but more detailed results of statistical analyses are provided in Supplementary Material (Table S1).

## Results

### Head Twitch Response

**FIGURE 1:**
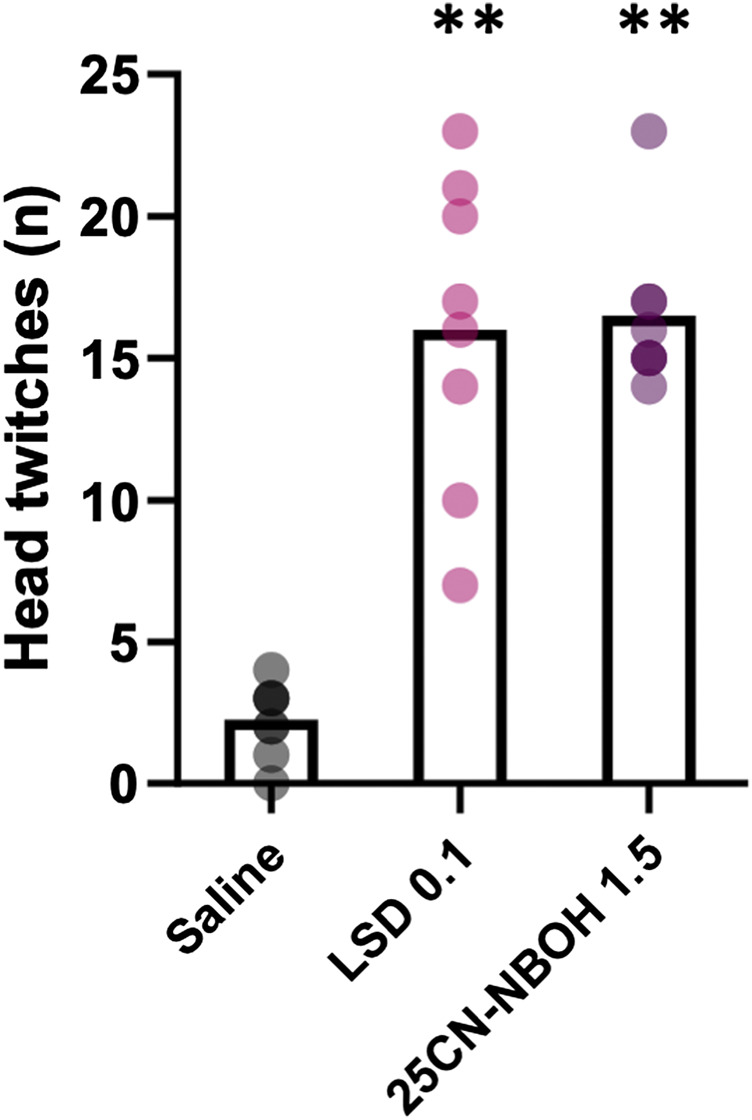
Number of head twitch responses observed during the first 10 min after administration of saline, 0.1 mg/kg LSD, and 1.5 mg/kg 25CN-NBOH (repeated administration, n = 8). The data are shown as means with individual values depicted by the dots. Statistically significant differences in comparison to saline shown as **p < 0.01.

The tested doses of LSD and 25CN-NBOH significantly increased the number of head twitches in comparison to saline during the first 10 min after administration (Figure 1; χ^2^ (2) = 12.00, *p* = 0.002, saline *vs* LSD 0.1 mg/kg: z = -1.50, *p* = 0.008, d = 0.809, saline *vs* 25CN-NBOH 1.5 mg/kg: z = -1.50, *p* = 0.008, d = 0.809) with large effect sizes.

### Mouse Iowa Gambling Task

**FIGURE 2:**
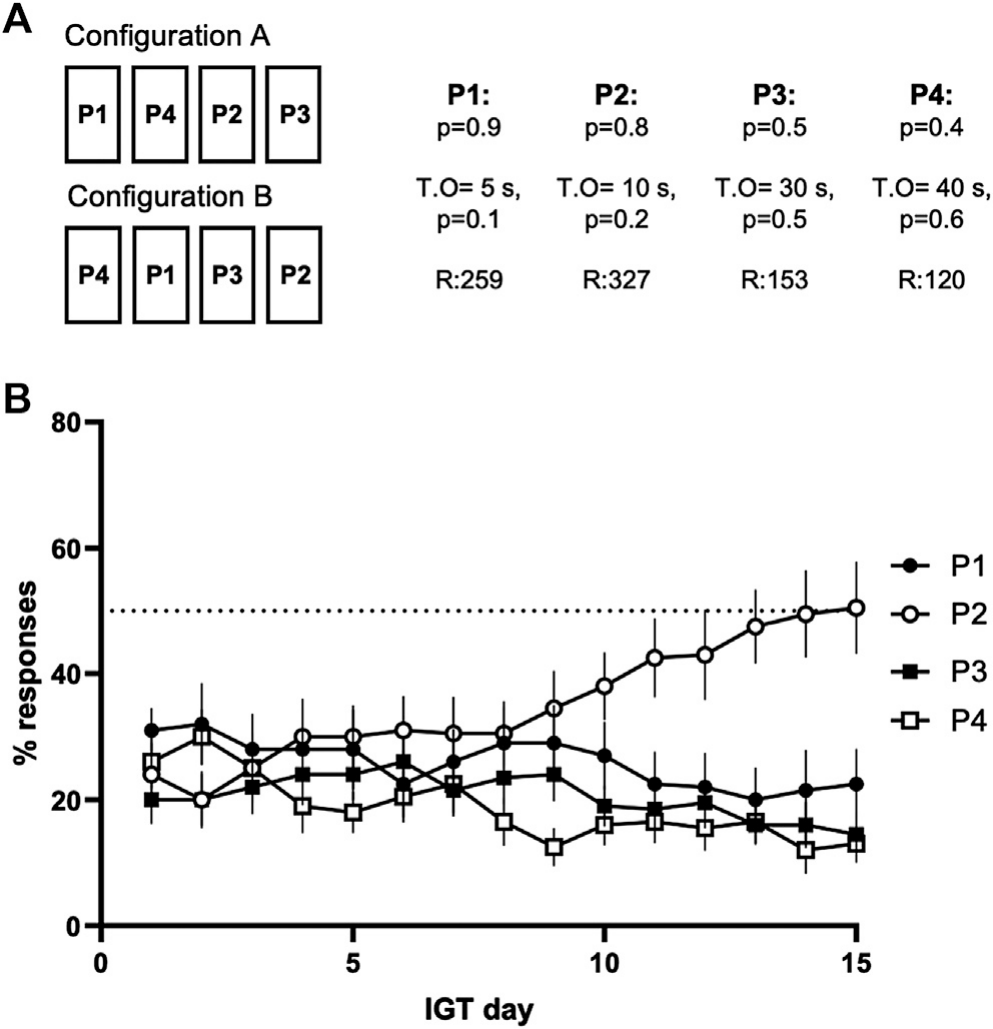
**(A)** The four active panels had unique reward-punishment contingencies, and the two different configurations, A and B, were used in different chambers. The panels are named after the number of sucrose rewards each of the panels gave. The first p-number indicates the probability of the reward. The punishments were given as time-out periods, the probability and the length of the time-out rising together with the reward (T.O for the time-out in seconds, *p* for the probability of the punishment). The R-numbers indicate the maximum number of reinforcements of each panel if it was chosen exclusively during the session **(B)** The development of option selection during the first 15 days of the Iowa Gambling Task phase, prior to the drug treatment sessions. Data shown as mean ± SEM.

After 15 days of the gambling task, the mice showed a stable performance with no significant differences in selecting an option over four consecutive days (P1: χ^2^ (3) = 0.729, *p* = 0.866, P2: χ^2^ (3) = 4.518, *p* = 0.211, P3: χ^2^ (3) = 1.739, *p* = 0.628, P4: χ^2^ (3) = 4.189, *p* = 0.242). The mice also exhibited a significant preference for selecting an option (χ^2^(3) = 8.867, *p* = 0.031), choosing P2 more often than P4 (z = 1.278, *p* = 0.018), but showed no statistically significant preference in comparison to the two other options (P2 *vs* P1: z = -0.722, *p* = 0.560, P2 *vs* P3: z = 0.667, *p* = 0.728; Figure 2B).

#### Amphetamine

##### Option Selection

**FIGURE 3:**
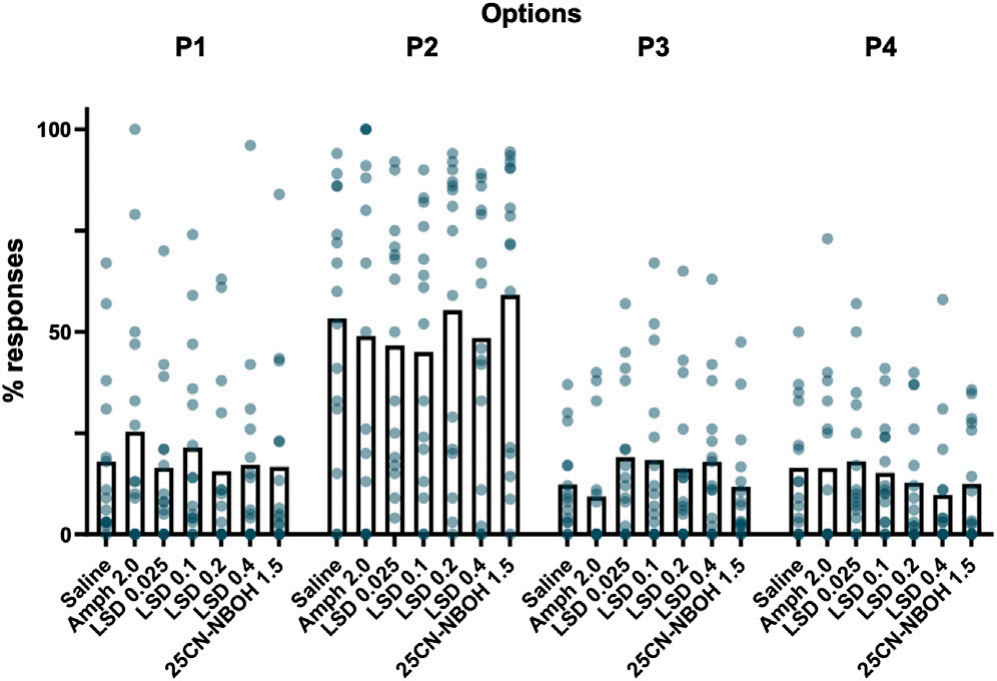
Mean percentages of choices in each reinforcement option respectively during the different drug treatment sessions of the Iowa Gambling Task phase. None of the acute drug treatments showed statistically significant effects on the reward-driven option selection. The dots depict values of individual mice.

Administration of amphetamine did not change the option selection in comparison to saline; there were no significant changes in favorable choices (Favorable %: χ^2^(6) = 11.190; *p* = 0.083). While there was a slight increase in P1 responses and a decrease in P2 responses (Figure 3) as previously reported (van Enkhuizen et al., 2013), the main effect differences were not significant (P1%: χ^2^(6) = 7.018, *p* = 0.319; P2%: χ^2^(6) = 10.123, *p* = 0.120). The choices for P3 and P4 remained virtually identical between saline and amphetamine (P3%: χ^2^(6) = 17.047, *p* = 0.009, saline *vs* amphetamine z = 1.400, *p* = 1.000, d = 0.53; see Supplementary Material Table S1 for details; P4%: χ^2^(6) = 6.389, *p* = 0.381).

##### Response Execution

**FIGURE 4:**
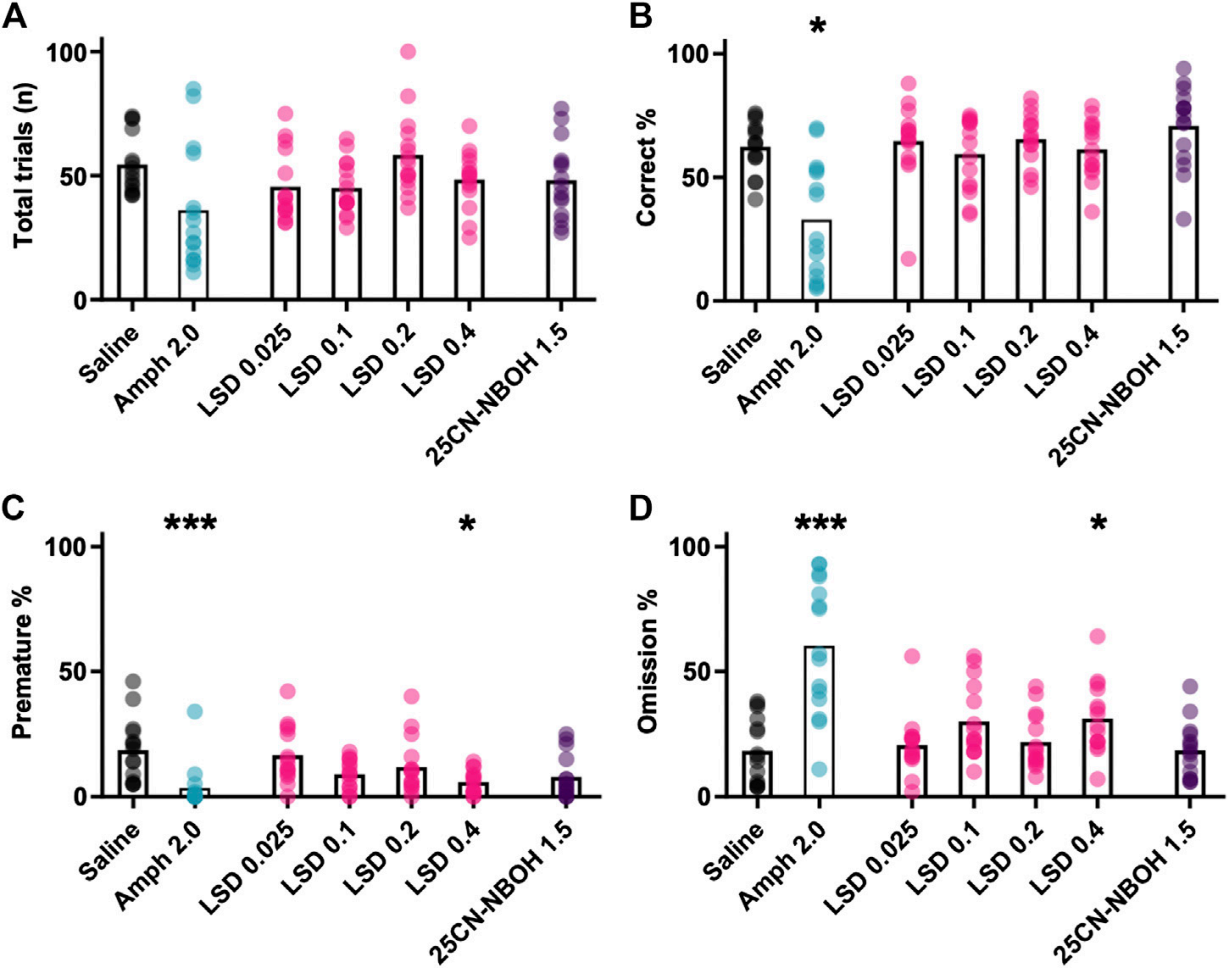
Mean effects of different drug treatments on performance in the Iowa Gambling Task phase. The data show the mean and the individual values of the total number of initiated trials (**A**), the percentage of correctly timed responses (Correct % (**B**)), the percentage of prematurely timed responses (Premature % (**C**)), and the percentage of completely omitted trials (Omission % (**D**)). Statistically significant differences in comparison to saline shown as ***p < 0.001 and *p < 0.05.

Amphetamine decreased the total number of trials the mice initiated during the session in comparison with saline (Figure 4A; mean ± SEM: saline 54 ± 3, amphetamine 36 ± 6). Despite the large effect size, the observed difference did not reach the set limit of statistical significance (χ^2^(6) = 17.682, *p* = 0.007, saline *vs* amphetamine z = 2.267, *p* = 0.085, d = 0.91). Considering the total number of trials, amphetamine administration decreased the correct responses (Figure 4B; χ^2^(6) = 34.882, *p* < 0.001; saline *vs* amphetamine z = 2.467, *p* = 0.037, d = 1.0) and premature responses (Figure 4C; χ^2^(6) = 36.824, *p* < 0.001; saline *vs* amphetamine z = 3.900, *p* < 0.001, d = 2.03), while markedly increased the omission percentage (Figure 4D; χ^2^(6) = 39.943, *p* < 0.001; saline *vs* amphetamine z = –3.800, *p* < 0.001, d = 1.93).

#### Lysergic Acid Diethylamide

##### Option Selection

None of the tested LSD doses caused any significant changes in option selection when compared to saline. The proportion of favorable choices remained the same (Favorable %: χ^2^(6) = 11.190, *p* = 0.083) and the changes in choices were similar in size when compared with amphetamine; none of the differences were statistically significant (Figure 3; P1%: χ^2^(6) = 7.018, *p* = 0.319; P2%: χ^2^(6) = 10.123, *p* = 0.120; P3%: χ^2^(6) = 17.047, *p* = 0.009, saline *vs* LSD 0.025–0.4 mg/kg -1.3 < *z* < -0.4, *p* = 1.00, d < 0.53; P4%: χ^2^(6) = 6.389, *p* = 0.381).

##### Response Execution

The tested LSD doses had no clear effect on the total number of initiated trials (Figure 4A (χ^2^(6) = 17.682, *p* = 0.007; saline *vs* LSD 0.025–0.4 mg/kg -0.433 < *z* < 1.733, *p* > 0.588; d < 0.68). The same was true with the correct responses (Figure 4B; χ^2^(6) = 34.882, *p* < 0.001; saline *vs* LSD 0.025–0.4 mg/kg −0.667 < *z* < 0.033, *p* = 1.00; d < 0.25). The highest dose of LSD (0.4 mg/kg) significantly decreased the premature responses with a large effect size (Figure 4C; χ^2^(6) = 36.824, *p* < 0.001; saline *vs* LSD 0.4 mg/kg z = 2.700, *p* = 0.013, d = 1.13) and also increased the omissions with a large effect size (Figure 4D; χ^2^(6) = 39.943, *p* < 0.001, saline *vs* LSD 0.4 mg/kg z = –2.667, *p* = 0.015, d = 1.15). The lower doses of LSD showed no significant effects on the corresponding parameters (Figures 4C,D; premature [saline *vs*]: LSD 0.025–0.2 mg/kg 0.333 < *z* < 1.11, *p* = 1.000, d < 0.42; omissions [saline *vs*]: LSD 0.025–0.2 mg/kg −2.2 < *z* < −0.333, *p* > 0.111, d < 0.88).

#### 25CN-NBOH

##### Option Selection

Similar to LSD, the tested dose of 25CN-NBOH did not affect option selection with neither the proportion of favorable choice (Favorable %: χ^2^(6) = 11.190, *p* = 0.083) nor the individual choices showing any statistically significant changes (Figure 3; P1%: χ^2^(6) = 7.018, *p* = 0.319; P2%: χ^2^(6) = 10.123, *p* = 0.120; P3%: χ^2^(6) = 17.047, *p* = 0.009, saline *vs* 25CN-NBOH: z = 0.067, *p* = 1.00, d = 0.003; P4%: χ^2^(6) = 6.389, *p* = 0.381).

##### Response Execution

The tested dose of 25CN-NBOH did not significantly alter behavior during the gambling task session, with no statistically significant changes observed in total number of trials (Figure 4A; χ^2^(6) = 17.682, *p* = 0.007, saline *vs* 25CN-NBOH: z = 1.033, *p* = 1.00, d = 0.38), correct responding (Figure 4B; χ^2^(6) = 34.882, *p* < 0.001, saline *vs* 25CN-NBOH: z = -2.00, *p* = 0.236, d = 0.80), premature responding (Figure 4C; χ^2^(6) = 36.824, *p* < 0.001; saline *vs* 25CN-NBOH: z = 2.267, *p* = 0.085, d = 0.91), or in omission rate (Figure 4D; χ^2^(6) = 39.943, *p* < 0.001, saline *vs* 25CN-NBOH: z = -0.333, *p* = 1.00, d = 0.00).

## Discussion

In this study, we explored the effects of acute administration of the psychedelic drug LSD on goal-directed decision making and related behaviors in mice using the mouse version of the Iowa Gambling Task. We chose the timing of the LSD and 25CN-NBOH administration such that the onset of the effects expressed as head twitch responses would overlap with the gambling task. Based on earlier reports, the increase in head twitches is apparent within minutes after administration, peaking at approximately 5–7 min after administration, and remaining enhanced for more than 30 min, the time course of which therefore overlapped with the entire gambling task session (Halberstadt and Geyer, 2013; Buchborn et al., 2018). As similar head twitch responses are not induced by amphetamine, its administration schedule was based on a prior report of its effects in the Iowa Gambling Task (van Enkhuizen et al., 2013).

When assessing the reported results, several limitations should be considered. Due to the exploratory nature of the study, the number of tested animals was small. Animals of only one sex were used, treatments were not counterbalanced, and analyses were not blinded. As we did not have access to facilities with a reversed light cycle, the mice were tested during their inactive period. While the performance of the tested animals resembled previous observations (Zeeb et al., 2009), the light phase during the experiment is known to affect the behavioral readouts in general (Richetto et al., 2019). It should also be noted that several different forms of the rodent Iowa Gambling Task have been developed over the years (de Visser et al., 2011). From these, a newer paradigm based on a single-session performance testing could be considered more similar to the original clinically used human version of the task (van Enkhuizen et al., 2014). From the perspective of clinically relevant translatability, the use of the single-session version might be preferable. However, a recent study showed that mice and humans perform very similarly in Iowa Gambling Task if the experimental parameters were harmonized between the species (Cabeza et al., 2020). This strengthens the face validity of non-conventional, multi-session versions of the task like the one used here.

In the present study, the development of decision making behavior at baseline before any drug administration followed the same pattern as in previous studies using the rodent Iowa Gambling Task (Zeeb et al., 2009; van Enkhuizen et al., 2013). Our mice learned to optimize their responses within 15 days, as shown by the increase in the ratio of the theoretically best option P2 (Figure 1B). Our results with d-amphetamine also closely resembled those reported earlier by van Enkhuizen et al. (2013). We found a similar decrease in the total number of responses and an increase in omissions (Figures 4A and 4D). We also found that amphetamine prominently reduced premature responses, which is opposite to earlier findings in rats showing increases in premature responding with several doses of amphetamine (Zeeb et al., 2009), but consistent with the findings in mice (van Enkhuizen et al., 2013). Our data with d-amphetamine on decision making showed a decrease in P2 and an increase in P1 (Figure 3), which is similar to earlier studies. However, this result was not significant and had a small effect size. The differences might be caused by the nature of amphetamine’s effects, which have been previously shown to be highly dependent on the environment and not directly dose dependent (Cardinal et al., 2000).

Based on our current data, the acute doses of LSD (with the exception of the greatest dose of 0.4 mg/kg) or of the more specific 5-HT_2A_ receptor agonist 25CN-NBOH did not cause marked changes in reward-driven decision making or in general functioning of mice in the Iowa Gambling Task. To ensure that the drugs used in the experiment worked properly, we assessed the induction of the head twitch responses. Both LSD and 25CN-NBOH increased the number of head twitches similarly as previously reported (Figure 2; Halberstadt and Geyer, 2013; Buchborn et al., 2018). Moreover, we observed head twitch responses during the gambling task trials after every LSD and 25CN-NBOH administration, indicating that the lack of effect was not caused by deficient drug doses. However, systematic quantification of head twitches inside the operant chambers was not possible in our setup.

Direct comparison of these effects to previous studies is not possible as, to our knowledge, this is the first reported investigation of 5-HT_2A_ receptor agonists in the rodent version of the Iowa Gambling task. Nevertheless, previous studies that investigated drugs that affect 5-HT_2A_ receptors have shown similar results to ours. Both M100907, a 5-HT_2A_ receptor specific antagonist, and the lithium-mimetic ebselen, known to dampen 5-HT_2A_-related intracellular signaling, failed to show any effects on the rodent gambling task in rats (Adams et al., 2017; Barkus et al., 2018). Similar results on decision making behaviors together with psychedelic drugs have also been observed in humans. For example, a recent publication by Pokorny et al. (2019) showed no effects on risky decision making in the Cambridge Gambling Task after acute administration of LSD. Earlier, the same group also reported that acute psilocybin did not cause any observable effects on moral decision making in healthy human volunteers (Pokorny et al., 2017). These results suggest that 5-HT_2A_ activation does not play an important role in reward-oriented decision making or risk taking, which is consistent with the idea that the serotonin system influences domains such as probabilistic reversal and social decision making (Rogers, 2011).

From the tested LSD doses, only the largest dose of 0.4 mg/kg caused any observable and statistically significant changes in general functioning during the gambling task, namely decreasing premature responding and increasing the omission rate (Figure 4C and 4D). While decreases in premature responding in test settings like this are usually interpreted as lower levels of impulsivity or as increased task accuracy, the concurrent increase in omitted trials would imply more gross changes in behavior. Pokorny et al. (2019) showed that LSD increases deliberation time, measured as the latency between cue presentation and betting, in the Cambridge Gambling Task in humans. A similar effect could explain the results shown here, as an increase in deliberation time could cause the mice to miss the opportunity to react to cues in due time while simultaneously decreasing premature responding. However, one would assume this effect to be visible also as decreases in the proportion of the correct responses since all the phases had a time limit; the lack of changes in this parameter therefore does not fully support this view. Results analogous to ours have been reported before: using the five-choice serial-reaction time task (5-CSRTT) with rats, Carli and Samanin (1992) showed an increase in omissions after a 0.1 mg/kg dose of LSD. Similarly, Fletcher et al. (2007) used several doses of the 5-HT_2A_ receptor high-affinity agonist 2,5-dimethoxy-4-iodoamphetamine (DOI) and observed both increases in omissions and statistically non-significant decreases in premature responding, also in 5-CSRTT with rats. In the latter study, the increases in omissions were, unlike in the present study, accompanied by decreases in the total number of responses. Furthermore, Fletcher et al. (2007) also observed increased latency to respond correctly and to collect the reward, which could be indicative of increased deliberation time. Nevertheless, these effects could stem from changes in a variety of cognitive processes. As acute administration of LSD has been shown to disrupt a wide range of executive functions beside risky decision making in humans (Pokorny et al., 2019), further research is needed to understand these effects better.

The decreases in premature responding caused by 0.4 mg/kg LSD contradict some of the existing literature on 5-HT_2A_ receptor function in premature responding and impulsivity. In general, an inverse relationship between serotonin signaling and impulsivity is considered to exist (Linnoila et al., 1983; Worbe et al., 2014). Within this framework, 5-HT_2A_ activity has been shown to positively correlate with increased impulsivity. For example, DOI has been shown to increase premature responding in 5-CSRTT (Koskinen et al., 2000a), and M100907 also reduces the number of premature responses in 5-CSRTT (Winstanley et al., 2004). 5-CSRTT and the Iowa Gambling Task are not identical tasks, but premature responding is considered an analogous measurement between the tests (de Visser et al., 2011). However, the relationship between 5-HT_2A_ activation and impulsivity is probably not so straightforward. Another study by Koskinen et al. (2000b) revealed that while increased premature responding remained, the effects of DOI were not thoroughly consistent if the ITI length was changed. In our experiment, the more specific 5-HT_2A_ receptor agonist 25CN-NBOH, which resembles DOI more than LSD in its receptor profile (Jensen et al., 2017), failed to elicit any statistically significant changes in premature response behavior (Figure 4C). Reports of DOI enhancing, instead of diminishing, premature responding in rats have also been published (Fletcher et al., 2007). Taken together, these results suggest that 5-HT_2A_ receptor activation is not directly responsible for changes in impulsive-like behavior, but the differences in study species, strains, training, and environment in general might be important contributing factors.

The highest dose of LSD used in the present study can be considered rather high in comparison to those commonly used in rodent experiments (see *eg* Parker, 1996; Meehan and Schechter, 1998; Frankel and Cunningham, 2002; Kyzar et al., 2016; Alper et al., 2018). This, together with the well-known diversity of molecular targets of LSD (Passie et al., 2008), invites consideration of the effects of the 0.4 mg/kg dose of LSD beyond 5-HT_2A_ agonism. The higher dose of LSD may also mediate effects through other receptors, for example 5-HT_1A_ and _2C_ or dopamine receptors, all of which have been implicated in impulsive behaviors (Dalley and Roiser, 2012). Further studies are needed to investigate this possibility.

To conclude, while the exploratory nature of our study warrants further research on the subject, our results imply that mice can perform previously learned, reward-driven decision-making tasks while under the acute influence of LSD at the commonly used range of doses. This is, of course, based on the idea that the head twitch response elicited by the drug is considered as a temporal proxy of its consciousness-altering effects (Halberstadt and Geyer, 2018; Halberstadt et al., 2020). This information could prove valuable in designing future behavioral experiments using mice and psychedelic drugs, especially when executive function-related aspects are being tested. Our current data also show some potential for translational value, as a similar lack of effect in a gambling task following acute LSD administration has been reported in human volunteers (Pokorny et al., 2019). However, several open questions remain, especially related to the effects of higher doses of LSD and the role of 5-HT_2A_ agonism in decision making and related behavioral domains. These questions warrant further research on this subject.

## Data Availability Statement

The datasets presented in this study can be found in online repositories. The names of the repository/repositories and accession number(s) can be found below: Figshare https://doi.org/10.6084/m9.figshare.12906692.

## Author Contributions

Conceptualization: LE, PH, EK. Formal analysis: LE. Investigation: LE, NK. Writing—original draft: LE. Writing—review & editing: LE, NK, PH, EK. Visualization: LE. Supervision: PH, EK. Funding Acquisition: LE, EK.

## Funding

This study was funded by The Finnish Foundation for Alcohol Studies, The Finnish Cultural Foundation, and The Academy of Finland (no: 1317399), and the publication of this article by the Helsinki University Library.

## Conflict of Interest

The authors declare that the research was conducted in the absence of any commercial or financial relationships that could be construed as a potential conflict of interest.
