# Supplementary material for: Acute Lysergic Acid Diethylamide Does Not Influence Reward-Driven Decision Making of C57BL/6 Mice in the Iowa Gambling Task
Source: Front Pharmacol. 2020 Dec 3;11:602770. doi: 10.3389/fphar.2020.602770 (PMC7745734; doi:10.3389/fphar.2020.602770)
Supplement: Supplementary file 1 [file table1.docx]

**Supplementary Table S1. Supplementary statistics table**

| **Figure** | **Parameter** | **n** | **Test** | **Test statistics** | **p-value** | **Cohen’s *d*** |
| --- | --- | --- | --- | --- | --- | --- |
| 2 | P1-% | 15 mice | Friedman’s ANOVA | χ^2^(6)=7.018 | 0.319 |  |
|  | P2-% | 15 mice | Friedman’s ANOVA | χ^2^(6)=10.125 | 0.12 |  |
|  | P3-% | 15 mice | Friedman’s ANOVA | χ^2^(6)=17.047 | 0.009 |  |
|  |  |  | Dunn’s pairwise comparison with Bonferroni correction | Saline vs. amphetamine  z=1.400  Saline vs. LSD 0.025  z=–1.133  Saline vs. LSD 0.1  z=–0.733  Saline vs. LSD 0.2  z=–0.400  Saline vs. LSD 0.4  z=–1.300  Saline vs. 25CN-NBOH  z=0.067  amphetamine vs. LSD 0.025  z=2.533  amphetamine vs. LSD 0.4  z=2.700 | 1.00  1.00  1.00  1.00  1.00  1.00  0.028  0.013 | 0.53  0.42  0.27  0.15  0.49  0.03  1.04  1.13 |
|  | P4-% | 15 mice | Friedman’s ANOVA | χ^2^(6)=6.389 | 0.381 |  |
|  | Favorable-% | 15 mice | Friedman’s ANOVA | χ^2^(6)=11.190 | 0.083 |  |
| 3 | Total trials | 15 mice | Friedman’s ANOVA | χ^2^(6)=17.682 | 0.007 |  |
|  |  |  | Dunn’s pairwise comparison with Bonferroni correction | Saline vs. amphetamine  z=2.267  Saline vs. LSD 0.025  z=1.467  Saline vs. LSD 0.1  z=1.733  Saline vs. LSD 0.2  z=–0.433  Saline vs. LSD 0.4  z=1.167  Saline vs. 25CN-NBOH  z=1.033  amphetamine vs. LSD 0.2  z=2.700 | 0.085  1.00  0.588  1.00  1.00  1.00  0.013 | 0.91  0.556  0.68  0.16  0.44  0.38  1.13 |
|  | Correct-% | 15 mice | Friedman’s ANOVA | χ^2^(6)=34.882 | 0.000 |  |
|  |  |  | Dunn’s pairwise comparison with Bonferroni correction | Saline vs. amphetamine  z=2.467  Saline vs. LSD 0.025  z=–0.467  Saline vs. LSD 0.1  z=–0.033  Saline vs. LSD 0.2  z=–0.667  Saline vs. LSD 0.4  z=0.033  Saline vs. 25CN-NBOH  z=–2.033  amphetamine vs. LSD 0.025  z=2.933  amphetamine vs. LSD 0.1  z=2.500  amphetamine vs. LSD 0.2  z=3.133  amphetamine vs. LSD 0.4  z=2.433  amphetamine vs. 25CN-NBOH  z=–4.500 | 0.037  1.00  0.588  1.00  1.00  1.00  0.004  0.032  0.001  0.043  0.000 | 1.01  0.17  0.01  0.25  0.01  0.8  1.27  1.03  1.39  0.99  2.88 |
|  | Premature-% | 15 mice | Friedman’s ANOVA | χ^2^(6)=36.824 | 0.000 |  |
|  |  |  | Dunn’s pairwise comparison with Bonferroni correction | Saline vs. amphetamine  z=3.900  Saline vs. LSD 0.025  z=0.333  Saline vs. LSD 0.1  z=2.033  Saline vs. LSD 0.2  z=1.133  Saline vs. LSD 0.4  z=2.700  Saline vs. 25CN-NBOH  z=2.267  amphetamine vs. LSD 0.025  z=3.567  amphetamine vs. LSD 0.2  z=2.767 | 0.000  1.00  0.209  1.00  0.013  0.085  0.000  0.010 | 2.03  0.12  0.80  0.42  1.13  0.91  1.72  1.17 |
|  | Omission-% | 15 mice | Friedman’s ANOVA | χ^2^(6)=39.943 | 0.000 |  |
|  |  |  | Dunn’s pairwise comparison with Bonferroni correction | Saline vs. amphetamine  z=–3.800  Saline vs. LSD 0.025  z=­­–0.333  Saline vs. LSD 0.1  z=–2.200  Saline vs. LSD 0.2  z=–0.933  Saline vs. LSD 0.4  z=–2.667  Saline vs. 25CN-NBOH  z=0.000  amphetamine vs. LSD 0.025  z=–3.467  amphetamine vs. 25CN-NBOH  z=3.467 | 0.00  1.00  0.111  1.00  0.015  1.00  0.00  0.00 | 1.93  0.12  0.88  0.35  1.15  0.00  1.64  1.64 |
| 4 | Head Twitch Responses | 8 mice | Friedman’s ANOVA | χ^2^ (2)=12.00 | 0.002 |  |
|  |  |  | Dunn’s pairwise comparison with Bonferroni correction | Saline vs.  LSD 0.1  z=­­–1.500  Saline vs. 25CN-NBOH  z=–1.500 | 0.008  0.008 | 0.809  0.809 |
|  | | | | | | |
